# Influence of the natural radon radiation on the spread of the COVID 19 pandemic

**DOI:** 10.1038/s41598-023-39705-2

**Published:** 2023-08-07

**Authors:** Mykhaylo Yelizarov, Olexandr Yelizarov, Iryna Berezovska, Malgorzata Rataj

**Affiliations:** 1 Natural Sciences Department, Kremenchuk Mykhailo Ostrohradskyi National University, Kremenchuk, Ukraine; 2 Department of Artificial Intelligence, University of Information Technology and Management in Rzeszow, Rzeszow, Poland; 3 Department of Cognitive Science and Mathematical Modeling, University of Information Technology and Management in Rzeszow, Rzeszow, Poland

**Keywords:** Environmental sciences, Natural hazards, Medical research

## Abstract

The statistics of COVID-19 accumulated in Ukraine show areas with a significantly lower incidence of diseases. The purpose of the study was to identify factors that could influence the pattern of the pandemic in a particular area. Within the study it was assumed that the level of health care is approximately the same throughout the country. Population density was considered the main factor influencing the dynamics of the spread of infection. To reduce the impact of changes in population density across regions, it was normalized by the average population density in the country. The normalization of statistics for the country resulted in a model in the form of a linear relationship between the normalized values of the number of COVID-19 cases in the region and the size of the region. Subsequent analysis of the graphical data made it possible to identify four regions with the lowest incidence of COVID-19. The geographical proximity of these regions Dnipro, Kherson, Vinnytsia and Kirovograd, indicates the presence of a common factor for them, not typical for the rest of Ukraine. Such a factor may be the location of 83% of Ukraine's uranium deposits in the territories around Kirovohrad. Radon is one of the decay products of uranium, so the population of these areas may experience increased exposure to radon. This noble gas has more than a century of medical use, in particular for pulmonary diseases, although there is still no consensus about its effectiveness and side effects. Considering that COVID-19 was often complicated by pulmonary diseases, it can be assumed that the geological specificity of these four regions of Ukraine had an impact on the course of the COVID-19 pandemic in their territories. The study findings are important in terms of further COVID-19 research and prevention strategies.

## Introduction

Globally, as of 28 February 2023, 758,390,564 cases of COVID-19 have been confirmed, including 6,859,093 deaths, reported to WHO. Of these, there were 5,389,439 confirmed cases and 111,235 deaths in Ukraine (https://covid19.who.int/).

In the fight against the Coronavirus Pandemic (COVID-19), the main focus is placed on developing new and improving existing vaccines and drugs. However insufficient efficiency of available treatment options for this viral infection and often pulmonary complications have fueled the interest for alternative therapy. As an example, low-dose radiation therapy can be mentioned. Its immunological perspective and treatment results are under consideration and some of them have been published^1^.

It should be mentioned that low-dose radiation may be of natural origin. Its adverse influence has been explored in many aspects^2^, but potential impact on the course of some diseases is not clear, although there are certain signs of this impact^3^.

One kind of natural radiation is produced by radon, a noble radioactive gas, which is one of the decay isotopes of uranium.

Radon therapy found its application many years ago^4–6^. Nevertheless, long indoor stay under increased radon irradiation which occurred during COVID-19 lockdowns was recognized as a risk factor for cancer disease^7^.

In Ukraine, radon was recognized as “the most significant dose forming factor” in the irradiation of the population^8^. Generally, the level of natural radiation of the population was estimated with effective doses according to the recommendations of the International Commission on Radiological Protection^9^ and the UNSCEAR dose coefficients^10^. An average annual effective dose of natural radiation includes the following components: an average annual effective dose due to indoor radon, an average annual effective dose of external gamma exposure produced by building materials and raw, an average annual effective dose due to radionuclides in drinking water^11^. This value was found to be equal to 3.5 mSv per year, with a substantial portion (72%) being formed by indoor radon—2.4 mSv per year. Dose values measured at ground floor in multistory buildings were 1.3–1.5. mSv per year, and they were about 1 mSv per year on upper floors. The weighted average effective dose from building materials is 0.23 mSv per year, which is lower than in previous years due to the introduction of new requirements for building materials. As an aside, it is worth mentioning drinking water which is another factor of the radon entry. Generalized results of the 20 years radon monitoring in Ukraine has showed that “for 11% of the tested water samples, 222Rn concentration exceeds the established state standard for drinking water (100 Bq/l)”^12^.

Health risks are assessed in many areas with high natural background radiation (HNBR), with China, Brazil, India and Iran being the most studied regions^13^.

In Yangjiang, China, an epidemiological study was carried out for 20 years in a HNBR area characterized by a uniform distribution of natural gamma radiation and great population stability. The comparison of this population with a control group of the same size in the neighboring area was based on examining about one million cumulative person-years in terms of cancer mortality. There was no increase in mortality in HNBR areas compared to the control group^14^.

One of the goals of a study^15^ conducted in Kohgiluyeh and Boyer-Ahmad Province, Iran, was to quantify cancer incident risk resulting from the background radiation in this province. The averaged over all cities percentage of excess lifetime cancer risks due to indoor exposure was found to be 4.6% for whole populations and 3% for adults.

Data of health effects in HNBR areas of Brazil was examined in a descriptive study^16^, and no formal epidemiological studies have been reported yet^13^. Mortality rates over the period 1991–2000 in the highly populated regions of Poços de Caldas and Araxà were compared with those in the entire Minas Gerais State. A standardized mortality ratio for cancers in Poços de Caldas and for non-cancer mortality in both regions was elevated. Chromosome aberrations were observed^17^.

The lack of data on mortality in the much smaller HNBR areas in Brazil comes to attention. Some explanation may be found in the statistical analysis made by Indian researchers^18^. They considered the data needed to build a response curve to low doses of radiation and concluded that a study base of at least 100,000 person-years is needed to ensure its statistical significance. Therefore, epidemiological studies in densely populated countries such as China and India seem to offer the best opportunity to study the effects of low levels of radiation at health risks.

The quality of the HNBR-exposed population study depends on meeting many requirements, including "appropriate study design and sufficient statistical power, and they must have available individual estimates of doses to specific organs from internal and external exposures, and individual information on known and possible risk factors for the diseases of interest"^13^. Limitations are also numerous; for example, the lack of well-documented health statistics and cancer rates in HNBR countries. This forced some researchers to declare that such studies can't provide definite explanations^19^.

However, the uncertainty of findings related to the health problems created by HNBR and the need for a clear assessment of the consequences were noted at an early stage of such studies as a challenge which is as great as ever. Expected health problems created by HNBR are hard to detect for a few reasons^20^: the health problems involved would exist to some degree irrespective of radiation exposure, other factors affect the incidence of such problems, the differences between normal background radiation levels and HNDR levels are not extreme.

Generally, the COVID-19 pandemic has revealed interesting phenomena and put many questions. For example, a number of COVID-19 cases in some regions of Ukraine was definitely lower (for example, Kirovograd, Kherson, Vinnytsia) than in other regions. These regions feature increased natural radiation because most of uranium deposits in Ukraine are located in these few regions.

This article focuses on the analysis of normalized system statistics on the development of COVID-19 to detect certain regularities and (or) anomalies of this process depending on the geographical features of a particular area of Ukraine.

## Method

**Figure 1:**
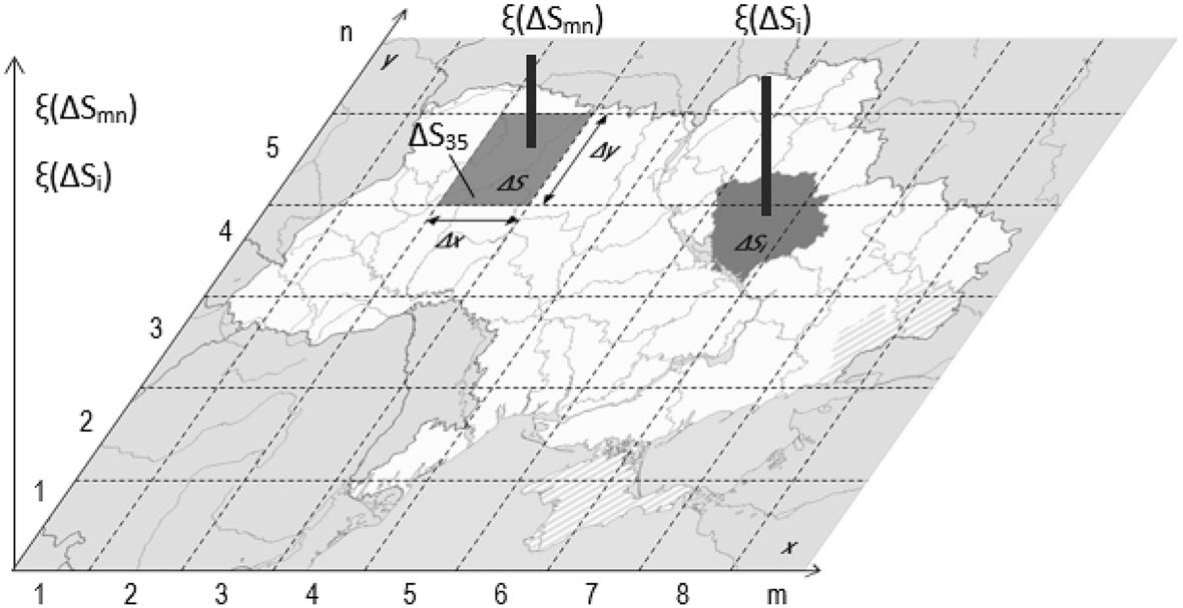
Graphical representation of the function ξ(x, y) when numbering areas of regular and irregular shape.

Let there be some value ξ (physical, economic, social) that characterizes a certain territory, for example, Ukraine. The value of ξ is essentially generalized—it is a value averaged or integrated over a particular area (for example, average annual precipitation over regions or a country's population). In our case, this value ξ should be examined on a local scale as the dependence ξ (x, y), where x and y are the coordinates of the area (Fig. 1). However, the Cartesian coordinate system can't be used in our case, since statistics (population density, morbidity, etc.) are not tied to the elements ΔS = (ΔxΔy), but to administrative units—regions, districts, cities. With this, the differentiation of the territory has to come down to the elements ΔS1 ≠ ΔS2 ≠ ΔS3 ≠ ….. ΔSi …… ≠ ΔSn, where ΔSi are the areas of regions, which should be compared by their parameters (population, population density, etc.). It means that the function ξ(x,y) with equal-sized elements ΔS = (ΔX ΔУ) has to be substituted by the function ξ(ΔS1 ≠ ΔS2 ≠ ΔS3 ≠ ….. ΔSi …… ≠ ΔSn) (Fig. 1).

### Mean value ξ (ΔSi) over the array ΔSi, deviation range, extrema

**Figure 2:**
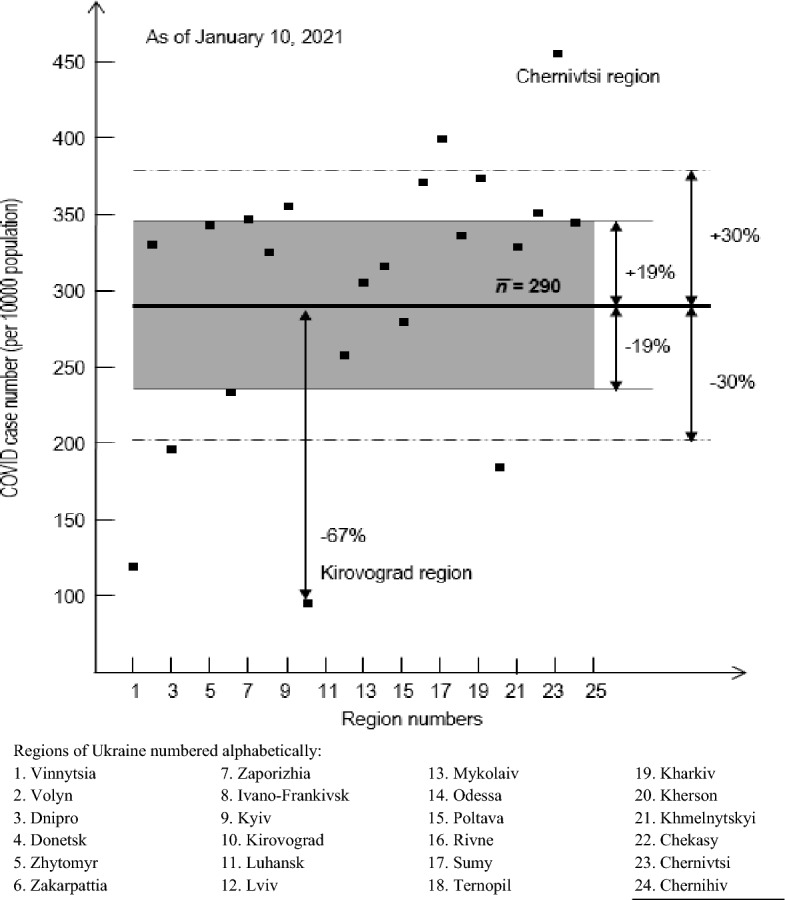
COVID-19 case numbers (per 10,000 population) over regions of Ukraine as of January 10, 2021.

One important clarification needs to be made here. It concerns the difference in the representation of the function ξ for regular ΔS = (ΔX ∙ ΔY) and irregularly shaped areas. In the first case, the row and column numbers (indices m and n) accurately indicate the location of the area, while in the second case, it is impossible to do this. Instead, the areas simply have to be numbered, each time specifying the principle of numbering (for example, alphabetically by the names of regions (Fig. 2).

The Public Health Center of the Ministry of Health of Ukraine (https://phc.org.ua/) has collected a large volume of statistics on COVID-19 pandemics in Ukraine (Coronavirus in Ukraine. Portal Minfin.com.ua. https://index.minfin.com.ua/ua/reference/coronavirus/ukraine/2021-01/)^21^.

Figure 2 shows COVID-19 case numbers (per 10,000 population) in each region of Ukraine as of January 10, 2021. Regions are numbered alphabetically.

The bold line in Fig. 2 presents the average case number (per 10,000 population) over regions n_average_. The shaded strip is a scatter range of the number of cases across regions within the limits of n_average_ + δ_average_, where δ_average_ is the average deviation in %, calculated in the standard way as (1):

1 $${\delta }_{average}=\frac{{\sum }_{i=1}^{K}(n-{n}_{average}) }{K}*100$$

Here K is the number of regions*.*

Points corresponding to values within the limits of n_average_ + δ_average_ will be considered to have "normal" deviations from the average value, and those that go beyond the specified limits—"special". It is the latter, in our opinion, that are of special interest to discover the main factors affecting the pandemic pattern.

In this research we will focus on specific features of the regions with the lowest number of cases to determine factors which may retard the pandemic progress**.**

Note that 14 regions out of 24 fell into the scatter range. Two regions have extreme deviations: Kirovograd—67% below n_average_ and Chernivtsi—59% above n_average_.

Though the analysis is based on the number of cases (per 10,000 population), the comparison of regions might be not very apt for some reasons: statistics on Kyiv region don't include the metropolis of Kyiv that is reasonable, but data related to Kharkiv, Dnipro, Odessa, Lviv are included in statistics on these regions; population density which is an important risk factor of COVID-19 varies across regions.

To take these conditions into account, we extend by one and a half times the scatter range to increase a number of regions with normal deviations and thus to highlight the most abnormal cases. The extended range corresponds to 30% deviation from the average value, it is represented by dotted lines. Four regions with lowest numbers of cases—Vinnytsia, Dnepro, Kirovograd and Kherson—are now below the bottom line of the scatter range. Note that they are adjacent to each other in Ukraine.

### Leveling the conditions for the infection spread across the regions

One of the important factors affecting the dynamics of the infection spread is the population density in a particular area. It, of course, is different in the regions of Ukraine and varies from 31 people/km^2^ in Chernihiv region to 155 people/km^2^ in Donetsk region, i.e. differs by 5 times. The average density is 73.3 people/km^2^. It should be noted that when calculating the average density value, inhabitants of Kyiv (where the intensity of human contacts between both locals and visitors is extremely elevated in comparison with other locations in the country) were excluded, but total populations of other regions were calculated taking into account people living in regional capitals. There are other circumstances not taken into consideration such as a different percentage of the rural population compared to the urban one or the situation with transport communications. More developed public transport seems to increase the intensity of human contacts, and urban population has more frequent social contacts than people living in rural areas. However, these details are secondary regarding the population density in a particular area which is considered as a main factor influencing the intensity of human contacts and hence the dynamics of the infection spread.

We normalize case numbers (per 10,000 population) across regions by average population density. Thus, the impact of varying population density on the dynamics of the pandemic spread can be reduced. This approach is expected to more clearly reveal other reasons explaining why the pandemic occurred in different patterns in different regions. Normalization coefficients are calculated as (2):

2 $$k=\frac{population\; density \;in \;a \;region}{average \;population \;density \;in \;Ukraine}$$

**Figure 3:**
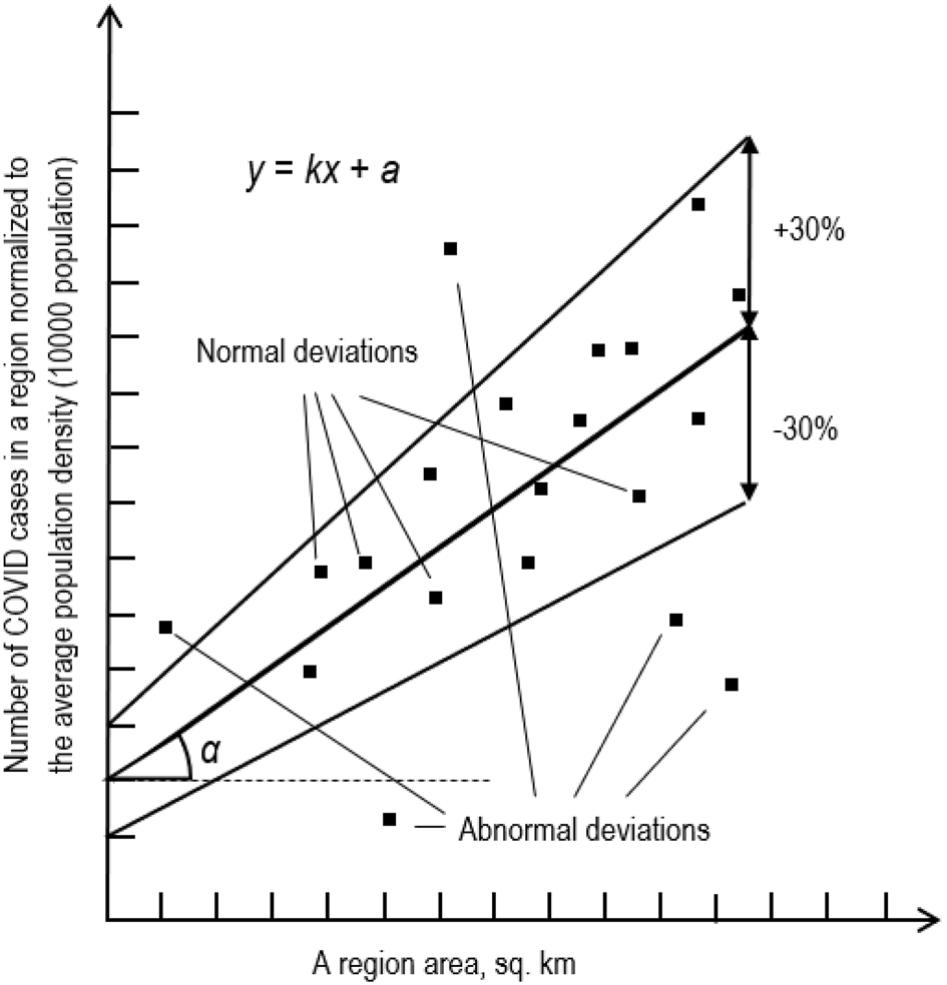
The predicted linear relationship between the case number (per 10,000 people) normalized to the average population density and region areas (a ≠ 0); scattered points forming the relationship are shown.

Normalizing the region population to the average population density in Ukraine results in a linear proportion between the normalized number of cases (per 10,000 people) in the region and the area of the region (Fig. 3).

Figure 3 illustrates how a theoretical model describing a relationship between the normalized number of cases (per 10,000 people) in the region and the region area is built. To be specific, the data referring to Ukraine were used to draw the picture. However, this approach can be applied to other countries as well.

The coefficient k in this case means the number of cases (per 10,000 population) per unit of area reached by the pandemic. Further, 10,000 km^2^ is used as a unit of area. Thus, a value of k is measured as (3):

3 $$\frac{number\; of \;cases \;in \;a \;region}{10,000 \;population*10,000 \;\text{km}^{2} \;of \;a \;region \;area}$$

The parameter *a* is measured in following units (4):

4 $$\frac{number \; of \; cases \;in \;a \;region}{10,000 \;population}$$

It determines the initial conditions of observation. *a* = *0*, if the number of cases is counted from the beginning of the pandemic, and *a ≠ 0,* if the number of cases is counted within a certain time period of the ongoing pandemic.

Obviously, the factors mentioned above that are not taken into account may influence the accuracy of this linear relationship. Let us estimate possible deviations from strict dependence.

Let's consider the worst case, namely, the scatter range which is extended up to 30% (Fig. 2). Therefore, the deviation of the points of this linear relationship should be less than 30%, because the case number (per 10,000 population) across regions is normalized by average population density. Thus, it can be used as a start approximation (Fig. 3).

Essentially, the straight line itself is built according to the argument and function values as some geometric averaging of statistics (Fig. 3). This approach determines the parameters of the straight line.

Thus, this linear relationship could serve as a baseline and model for further assessments, assuming that deviations from the straight line within 30% are quite acceptable. Otherwise, if points corresponding to certain regions do not fall into this range, these deviations are considered abnormal phenomena caused by the specific, compared to other regions, features of these particular regions.

## Results

**Figure 4:**
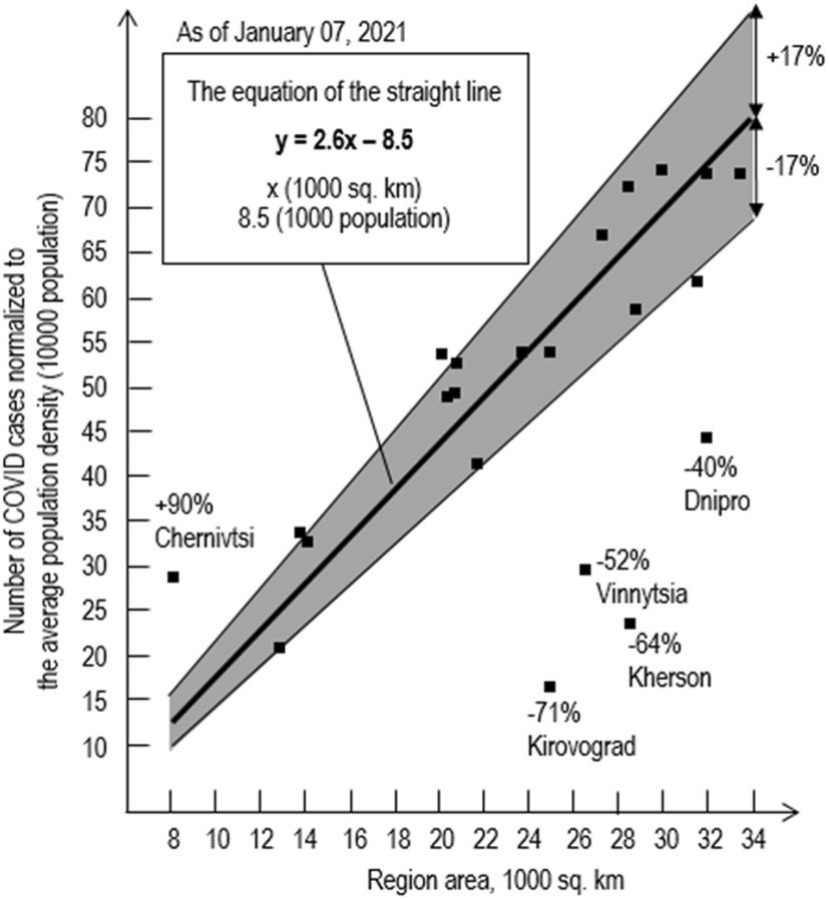
The number of COVID-19 cases normalized to the average population density of Ukraine dependent on the region area.

**Figure 5:**
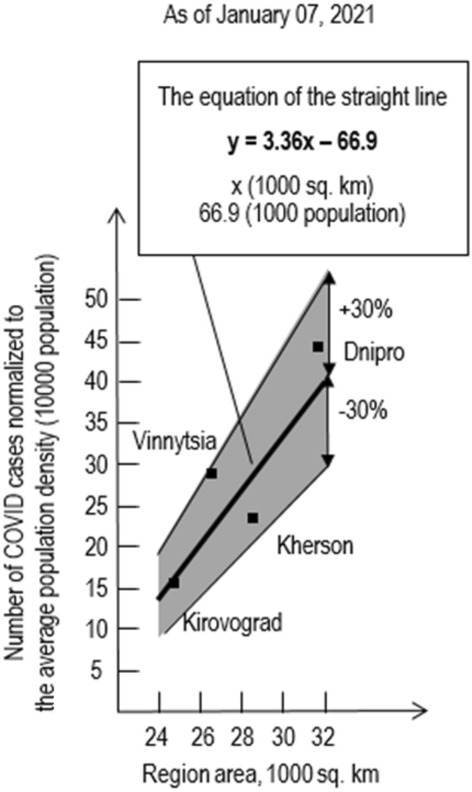
The linear relationship between the number of cases normalized to the average population density in Ukraine and area of regions with minimum numbers of cases (taken from Fig. 4).

The approach illustrated by the Fig. 1 was used to build linear relationships presented on Figs. 3, 4, 5.

To process statistics on the total of COVID-19 cases in Ukraine as of 07.01.21 (Fig. 4.), the equation of a straight line has the form (5):

5 $$y=2.6\frac{\left(1000 \; population\right)}{\left(1000 \;\text{km}^{2}\right)}x\left(1000 \;\text{km}^{2}\right)-8.5 (1000 \;population)$$

Please note that:

- 17 regions out of 22, are within the deviation range + − 17%, which is almost 2 times lower than the estimated 30%, and this may indicate a correct approach to the processing of statistics;
- five regions clearly fall out of this range, while one (Chernivtsi) has an extremely high number of cases, and four (Dnipro, Kherson, Vinnytsia and Kirovograd) have minimal incidence rates;
- the Chernivtsi region is outside of this research;
- further discussion will focus on possible reasons for the low incidence in mentioned above four areas.

The first to see is that they are located next to each other, which suggests the natural features of the area in which they are located.

The following conclusions and summarizing can be made from the above:

- these four regions form a certain united area with a special dynamic of the spread of the COVID-19 pandemic,
- if this is right, the linear relationship between the number of cases normalized to the average population density in Ukraine and area of abnormal regions can be generalized (Fig. 5).

It is clear that the equation of the resulting straight line will have other parameters:

6 $$y = kx + a$$

7 $${\text{y}} = {3}{\text{.36x}} - {66}{\text{.9}}$$

Figure 5 shows that this analysis provides the maximum deviation (Vinnitsa region) of 23% and can be considered adequate.

**Figure 6:**
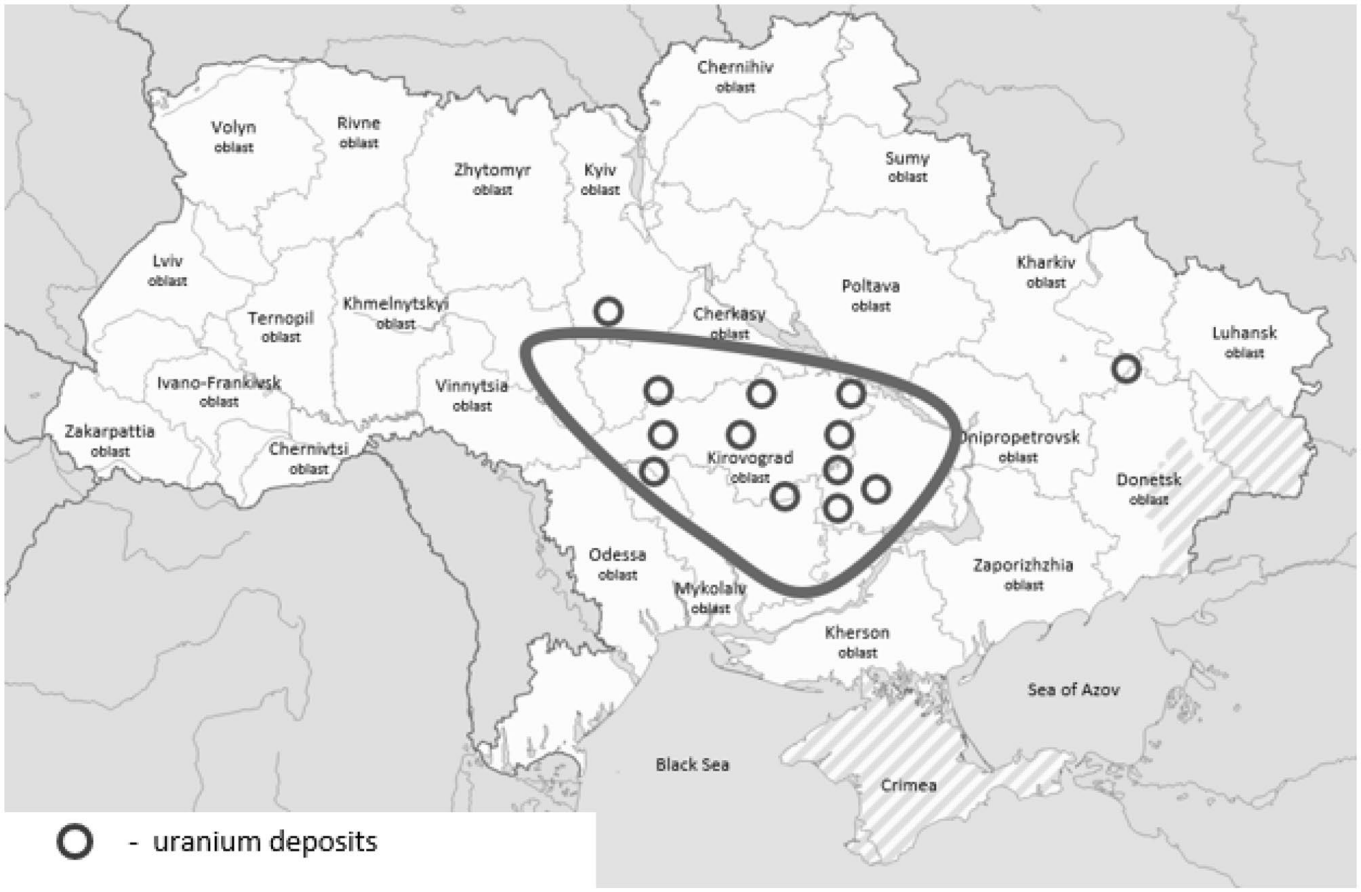
Outline of the territory of Ukraine with the lowest numbers of COVID-19 cases. Locations of uranium deposits are displayed accordingly to the map available at Buzinny and Soroka^20^.

"Abnormal" regions form a special area with a special linear relationship between the number of cases normalized to the average population density of Ukraine and the region areas. It is mapped as presented in Fig. 6.

## Discussion and conclusions

Normalizing the regional population density to the average population density of Ukraine made it possible to identify a number of regions with “abnormal” indicators of active cases of COVID-19.

“Abnormal” regions with low numbers of cases as of January 2021 form a united area (circled in Fig. 6).

To proceed with analyzing these specific features, we believe that:

- the quality of health care services in these regions does not differ from the quality in other regions;
- the fact that these regions are next to each other may indicate the uncommon physical and geological conditions of this area.

The territory of these regions circled in Fig. 6 belongs to the related basin of the Southern Bug, Ingulets and Ingul. It can be assumed that the river beds of this basin are rocks of the same type. Further, let's take into account that 83% of uranium deposits in Ukraine are concentrated in the Kirovograd region, where the number of COVID-19 cases is the lowest. It can be suggested that the rocks of the regions neighboring Kirovograd contain the same uranium, although in much less percentage. It is known that one of the decay isotopes of uranium is radon, a noble radioactive gas.

The list of recommended indications for radon treatment includes pulmonary diseases^19^.

Its clinical effects in respiratory and other diseases have been evaluated and discussed in many studies for example^22,23^. Radon therapy continues to be studied to gain medical evidence regarding its effectiveness and create a scientific basis to explain the controversy and the mechanism of radon treatment. It was shown that though radiation may increase production of free radicals, it activates their neutralization preventing expression of damaging actions of radicals. This may explain the beneficial effects of small doses of ionizing radiation^24^. Besides, small doses of ionizing radiation have a different mechanism of actions that induce responses which is not the same as the mechanism of high doses^25^. Radon or other gaseous sources are seen as a good alternative in the case when particular medications can be contraindicated in some patients^26^.

A recent study^27^ quantitatively evaluates the relation between the biological response caused by radon and the tissue/organ absorbed dose. Some new indications including inflammatory diseases and disorders caused by active oxygen were added to a recommended list. Study findings allowed to conclude that a small amount of active oxygen generated from radon activates the biological defense system; the amount of medicines may be reduced when drug therapy is combined with radon therapy.

Hence an increased exposure to radon in these four regions might be believed to affect the course of COVID-19 disease, especially in case of pulmonary manifestations. It is one of possible explanations why morbidity and mortality indicators in these regions are lower than in other regions of Ukraine.

We also take into account the fact that the population Covid-19 testing rate was similar in the four mentioned regions. It should be recognized that the available data is incomplete and fragmentary in Ukraine because the data processing was interrupted by the armed conflict in February 2022. However, a few sources provide evidence to suggest a similar testing rate. The overview made by the UNICEF^28^ shows that the average daily number of COVID-19 PCR tests by 100,000 inhabitants was around 100–130 at the end of 2020. Also, in the forecast developed by the National Academy of Science of Ukraine^29^ the average weekly number of COVID-19 PCR tests by 10,000 inhabitants was estimated as 100 in the same regions at the end of 2021.

At this time the level of vaccination in these regions was also similar and varied between 32 and 40%^30^.

The normalization approach followed by graphical analysis has provided important data on the probable dependence of a number of COVID-19 cases on geological conditions of a particular region. These findings may be of importance for COVID-19 research, development of healthcare services and prevention strategies.

The completeness of the analysis is limited by the lack of complete and reliable statistics from the four regions mentioned above. These regions are on the front line of the armed conflict. For this lack of more comprehensive, reliable and up-to-date data which are not currently available neither on the national nor local levels the most common and proven parametric methods of mathematical statistics cannot be used. However our next step will be refining the analysis in terms of mathematical statistics as soon as peace is restored and relevant statistics may be accessed.

## Data Availability

As it is indicated in the main text, our research is based on a large volume of statistics on COVID-19 pandemics in Ukraine collected by the Public Health Center of the Ministry of Health of Ukraine (https://phc.org.ua/). These datasets are available to general public and may be accessed at the website “Coronavirus in Ukraine. Portal Minfin.com.ua (https://index.minfin.com.ua/ua/reference/coronavirus/ukraine/2021-01/)^13^. Data on the population COVID-19 testing rate in Ukraine is available in “COVID-19 UKRAINE: 1 Bi-Weekly Situation Overview 9 Nov–6 Dec, 2020” at https://www.unicef.org/ukraine/media/9931/file and “NAS of Ukraine: Forecast of developing the COVID-19 pandemic in Ukraine for December 8–21, 2021 (infographic)” at https://tverezo.info/post/148771. Data on the level of COVID-19 vaccination in Ukraine is available in “Statistics of vaccination against coronavirus (COVID-19) in Ukraine as of 31.12.2021” at https://index.minfin.com.ua/ua/reference/coronavirus/vaccination/ukraine/2021-12-31/.
